# Dataset on the influence TPB predictors on environmental responsive behaviour amongst head teachers in the northern region of malaysia

**DOI:** 10.1016/j.dib.2021.106815

**Published:** 2021-01-30

**Authors:** Logeswari Uthamaputhran, Fais Ahmad, Hazlinda Hassan, Mathivannan Jaganathan

**Affiliations:** a Ministry of Education, Putrajaya, Malaysia; b School of Business Management, College of Busines, Universiti Utara Malaysia, 06010, Sintok, Kedah, Malaysia

**Keywords:** Environmental responsive behaviour, Environmental attitude, Environmental subjective norm, Environmental perceived behavioural control

## Abstract

The study examined the relationship between environmental attitude, environmental subjective norm, environmental perceived behavioural control, and school headteachers' environmental responsive behaviour. The population of the study consists of primary school headteachers in the northern region of Malaysia who are attached to the Ministry of Education (MoE), Malaysia. An online survey was used to collect the data of the study from 167 sampled respondents. While Theory of planned behaviour underpinned the study, the researcher employed explanatory, descriptive, and hypothesis testing quantitative strategies to explain the relationship. Smart PLS 3.0 and SPSS 21 were equally used to analyse the data. The result of the data analysis revealed that environmental attitude, environmental subjective norm, and environmental perceived behavioural control significantly influence school headteachers' environmental responsive behaviour.

**Specifications Table**

**Table:** 

Subject	Organisational behaviour and Human Resource Management
Specific subject area	Environmental Responsive Behaviour (ERB)
Type of data	Table & Figure
How data were acquired	Online Survey. The questionnaire is provided as a supplementary file.
Data format	Raw analysis, descriptive, statistical
Parameters for data collection	To participate in this survey as a selected sample, the respondent should be a headteacher of primary schools (national (Malay), national (Chinese), national (Tamil).
Description of data collection	Three hundred questionnaires were distributed through online survey (computerised self-administrated questionnaire). 167 usable questionnaires were returned for the analysis.
Data source location	The data of the study were collected from the Northern region of Malaysia (Perlis, Kedah, Penang, and Perak).
Data accessibility	Data is provided with this article. Data can be accessed via https://data.mendeley.com/datasets/b3n3wk5p7g/2

**Value of the Data**

• The data presented will enable education policymakers to have a better understanding and insights into what causes better environmental responsive behaviour amongst school headteachers.
• Ministry of Education can leverage the data for decision making and the implementation of new policies regarding environmental responsive behaviour amongst school headteachers.
• The analysis of this data can give valuable insights into the roles which the Ministry of Education plays in enhancing environmentally responsive behaviour amongst school headteachers.
• The data can be used to enlighten researchers on the influence of TPB predictors on environmental responsive behaviour amongst headteachers

## 1 Data Description

The data analysis begins by establishing detailed information about the TPB predictors. The article describes data obtained amongst headteachers from the northern region of Malaysia. Data were gathered through a computer self-administered online survey. The questionnaire of the study is provided as a supplementary file. Additionally, the raw data file is attached to the article as a supplementary document. Table 1 shows that 167 data were analysed. Table 1 equally exhibits the demographic details of headteachers and primary schools in Malaysia. This includes the headteachers` gender, age, ethnicity, experience at current school, total years of experience, highest education qualification, job training, school location/states, school area, school types, number of students, and employees in the school. A total of 167 responses representing 69 (41.3%) are males, while 98 (58.7%) are females. Regarding the age, 125 (74.9%) headteachers are within the age bracket of 51–60, 34 teachers (20.4%) are within 41–50 years of age and 8 (4.8%) headteachers fall into the category of 31–40 years age group. Regarding the ethnic group, the majority (49.7%) of the headteachers are Malay, followed by Chinese (37.7%), Indian (11.4%), and others (2.4%). Concerning the experience at current school, most of the headteachers have more than one but less than a two-year experience. Regarding total years of experience, 90 headteachers (53.4%) have two years’ experience while only 1 (0.6%) possesses eight years’ experience.

**Table 1:** Demographic of participants ((*N* = 167).

Demographic variables	Category	Frequency	Percentage
**Gender**	Male	69	41.3
	Female	98	58.7
**Age**	31–40	8	4.8
	41–50	34	20.4
	51–60	125	74.9
**Ethnic**	Malay	83	49.7
	Chinese	63	37.7
	Indian	19	11.4
	Others	4	2.4
**Experience at current school**	1	25	15
	2	131	78.4
	3	5	3
	4	1	0.6
	5	1	0.6
	6	2	1.2
	7	1	0.6
	8	1	0.6
**Total number of years of experience**	1	9	5.4
	2	90	53.4
	3	39	23.4
	4	16	9.6
	5	5	3.0
	6	4	2.4
	7	3	1.8
	8	1	0.6
**Highest education qualification**	Diploma in Education	37	22.2
	Bachelor's degree	121	72.5
	Master's degree	8	4.8
	Ph.D degree	1	0.6
**School location/ States**	Perlis	7	4.2
	Kedah	57	34.1
	Penang	20	12.0
	Perak	83	49.7
**School area**	Urban	53	31.7
	Rural	114	68.3
**School types**	National (Malay)	89	53.3
	National (Chinese)	62	37.1
	National (Tamil)	16	9.6
**Number of students in your school?**	Less than 100	33	19.8
	101–300	65	38.9
	301–600	43	25.7
	601–900	16	9.6
	901–1200	9	5.4
	More than 1200	1	0.6
**Number of employees in your schools**	1–20	47	28.1
	21–40	64	38.3
	41–60	33	19.8
	61–80	17	10.2
	81–100	4	2.4
	101–120	1	0.6
	121–140	–	–
	141–160	–	–
	161–180	–	–
	181–200	1	0.6

Furthermore, regarding the education qualification, 121 (72.5%) headteachers have Bachelor's Degree, followed by Diploma, 31 (22.2%), 8 (4.8%) Master's Degree and only 1 (0.6%) possesses PhD Degree. Regarding the school location, data were gathered from 4 different states, namely, Kedah 57 (34.1%), Perlis 7 (4.2%), Penang 20 (12.0%), and Perlis 7 (4.2%). Concerning the school area, 114 (68.3%) schools were located in the rural areas while 53 (31.7%) were from the urban areas. The school types include 89 (53.3%) National (Malay) (Chinese) 62 (37.1%) and National (Tamil) 16 (9.6%). Concerning the number of students in the schools, 65 (38.9%) of headteachers responded that their schools have 101–300 students, followed by 43 (25.7%) with 301–600 students, 33 (19.8%) with less than 100 students, 16 (9.6%) with 601–900 students, 9 (5.4%) with 901–1200 and 1 (0.6%) with more than 1200 students. Regarding the number of employees in the school, 64 (38.3%) schools have 21–40 employees while only one school has 101–120 and 181–200 employees.

### 1.1 Measurement model

Table 2 presents the composite reliability and average variance extracted (AVE) of the independent variables (environmental attitude, environmental perceived behavioural control, environmental subjective norm) and the dependant variable (environmental responsive behaviour). The composite reliability values of the four reflective latent constructs ranged from 0.897 to 0.948 and which exceeded the recommended cut-off value of 0.7. Besides, AVE values of all the latent constructs are greater than the acceptable threshold of 0.5, and the values are in the range of 0.509 and 0.672. Table 3 exhibits the square root of the average variance extracted of each variable and illustrates that all the square root of the AVE values was larger than other correlation values amongst the latent variables. This indicated that the measures are discriminant.

**Table 2:** Composite reliability and Average Variance Extracted (AVE) of variables.

	Composite Reliability	Average Variance Extracted (AVE)
Environmental Attitude (ATTD)	0.902	0.509
Environmental Perceived Behavioural Control (PBC)	0.948	0.672
Environmental Responsive Behaviour (ERB)	0.907	0.554
Environmental Subjective Norm (SUBJNORM)	0.897	0.533

**Table 3:** Square root of the average variance extracted.

	ATTD	PBC	ERB	SUBJNORM
**ATTD**	**0.714**			
**PBC**	0.619	**0.820**		
**ERB**	0.650	0.680	**0.744**	
**SUBJNORM**	0.571	0.698	0.556	**0.730**

Bold indicates to highlight that diagonal values are higher than other values.

### 1.2 Structural model

**Fig. 1:**
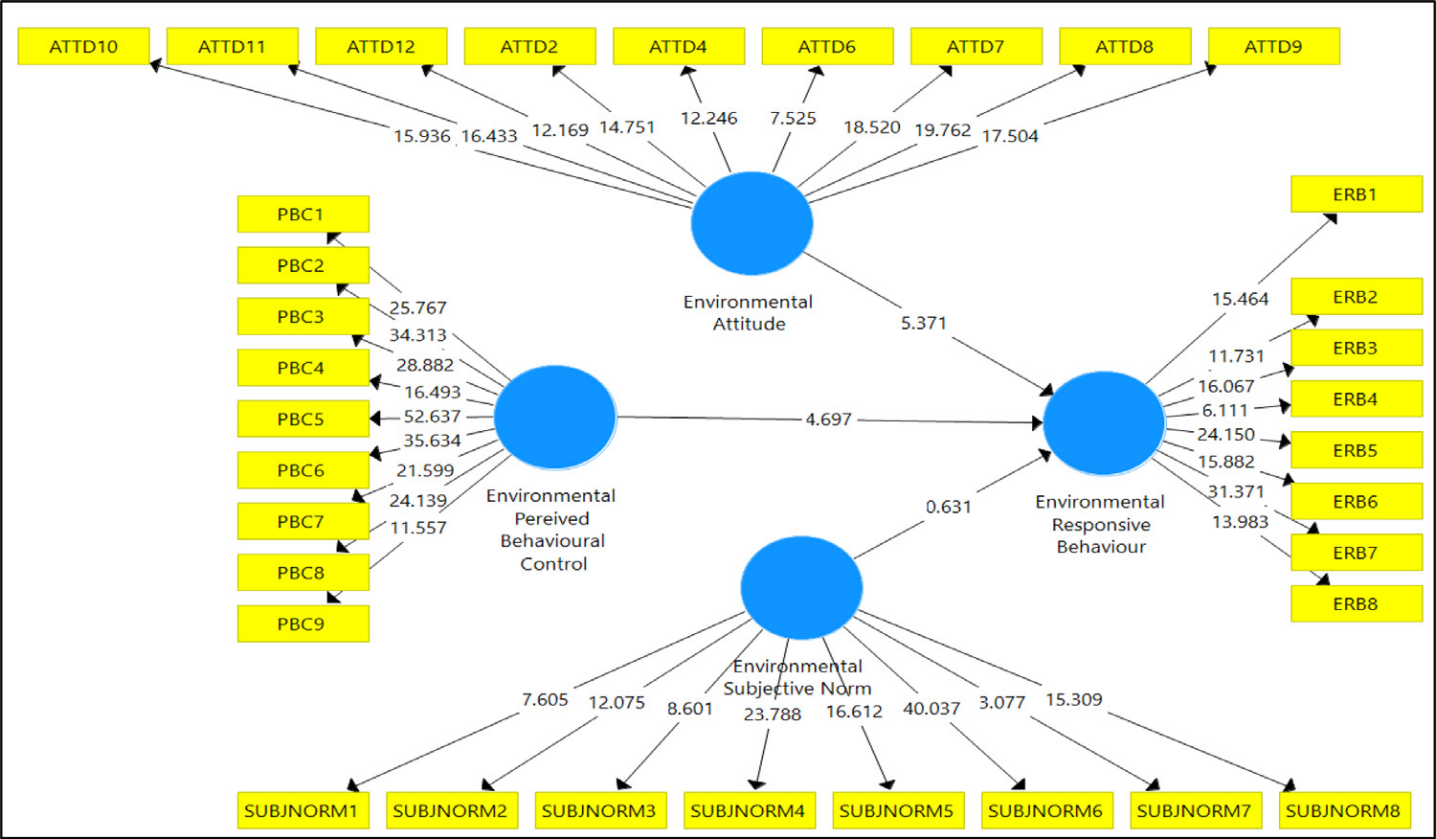
Structural Model.

Table 4 and Fig. 1 demonstrated the structural model that reflects the existence of significant relationships amongst related variables measured with path coefficient. The finding illustrated that environmental responsive behaviour (ERB) significantly influenced environmental attitude (ATTD) (*B* = 0.357 and *p* < 0.05) and environmental perceived behavioural control (PBC) (*B* = 0.417 and *p* < 0.05). However, this research found an insignificant result between environmental subjective norm (SUBJNORM) (*B* = 0.062 and *p* < 0.1) and environmental responsive behaviour.

**Table 4:** Path coefficient of the variables.

	Std Beta	Std. Error	t-value	Result
**ATTD -> ERB**	**0.357**	**0.066**	**5.371**	Supported
**PBC -> ERB**	**0.417**	**0.089**	**4.697**	Supported
**SUBJNORM -> ERB**	0.062	0.098	0.631	Supported

Note: PLS estimation results (*n* = 167, **p < 0.05, *p < 0.1).

Bold indicates to highlight the strongly supported results.

Additionally, the effect size was calculated by following Cohen's effect size estimation [1]. Effect size is considered small, medium, and large if the values are 0.02, 0.15, and 0.35, respectively. Therefore, the effect size was examined and reported in Table 5. Besides the path coefficient, the effect size can be evaluated to determine the respective impact of different variables in one model. In our case, Table 5 showed that environmental attitude (ATT), and environmental perceive behavioural control (PBC) have medium effect sizes of 0.164 and 0.170 respectively while environmental subjective norm (SUBJNORM) has a relatively small effect size of 0.004

**Table 5:** Effect size.

	Effect Size	Remark
**ATTD -> ERB**	0.164	medium
**PBC -> ERB**	0.170	medium
**SUBJNORM -> ERB**	0.004	Small

## 2 Experimental Design, Materials and Methods

Primary school headteachers from the northern region of Malaysia participated in this survey. These headteachers are considered as top managers in their respective schools, even though they are ranked as middle-level management in Malaysia's educational organisational hierarchy. They are also responsible to the district, state, and the Ministry for school performance; therefore, they are considered as the heart of device for school transformation and improvement. Three hundred questionnaires were distributed online to all the affected schools, while 167 usable questionnaires were returned for analysis. The data were then coded and analysed with SPSS-21 and SmartPLS 3.0. by taking into consideration the descriptive and statistical parameters of the variables.

## Ethics statement

Official permission was obtained from Education Planning and Research development (EPRD) as an authorised unit under the Ministry of Education, Malaysia before data collection from the primary schools. The online questionnaire was completely anonymous and did not contain any information that could identify the respondents as the respondents were offered the opportunity to stay anonymous, and their responses were treated confidentially.

## CRediT Author Statement

**Logeswari Uthamaputhran:** Conceptualisation, Data curation, Methodology, and Writing; **Fais Ahmad:** Conceptualisation; **Hazlinda Hassan:** Methodology and Writing; **Mathivannan Jaganathan:** Writing - review & editing.

## Declaration of Competing Interest

The authors declare that they have no known competing financial interests or personal relationships which have or could be perceived to have influenced the work reported in this article.

## Appendix Supplementary materials

Image, application 1
