# Supplementary material for: Dataset on the influence TPB predictors on environmental responsive behaviour amongst head teachers in the northern region of malaysia
Source: Data Brief. 2021 Jan 30;35:106815. doi: 10.1016/j.dib.2021.106815 (PMC7873344; doi:10.1016/j.dib.2021.106815)
Supplement: Supplementary file 1 [file mmc1.docx]

# SECTION A

**Following are some personal questions and statement related to your school background. Please complete and mark (X) the relevant information.**

# Please tick (/) in the appropriate box

| 1. | **Gender**  Male | Female |  |
| --- | --- | --- | --- |
| 2. | **Age** |  |  |
|  | Below 30 | 41-50 | 61 and above |
|  | 31- 40 | 51-60 |  |
| 3. | **Ethnic** |  |  |

Malay

Chinese

Indian

Others, *please specify*

# Experience at current school

| **Years** | **Mark X** |
| --- | --- |
| 1 |  |
| 2 |  |
| 3 |  |
| 4 |  |
| 5 |  |
| 6 |  |
| 7 |  |
| 8 |  |

# Total number of years of experience

| **Years** | **Mark X** |
| --- | --- |
| 1 |  |
| 2 |  |
| 3 |  |
| 4 |  |
| 5 |  |
| 6 |  |
| 7 |  |
| 8 |  |

# Highest education level

| **Mark X** | **Education Level** |
| --- | --- |
|  | Diploma of Education |
|  | Bachelor’s degree |
|  | Master’s degree |
|  | PhD degree |

# SECTION B

**Here are some statements related to your schools. Please tick (X) in the appropriate place and state the information where necessary about your schools.**

# School location

| **State** | **Mark X** |
| --- | --- |
| Perlis |  |
| Kedah |  |
| Pinang |  |
| Perak |  |

1. **School area:**

# (Tick X) (Tick X)

Rural

Urban

1. **School type**

| **Mark X** | **School type** |
| --- | --- |
|  | National |
|  | National (Chinese) |
|  | National (Tamil) |
|  | Religion |

# How many students are enrolled at your school?

| **Numbers of Students** | **Mark X** |
| --- | --- |
| less than 100 |  |
| 101 to 300 |  |
| 301 to 600 |  |
| 601 to 900 |  |
| 901 to 1200 |  |
| more than 1200 |  |

1. **State the total number of employees in your schools:**

| **Numbers of Employees** | **Mark X** |
| --- | --- |
| 1 to 20 |  |
| 21 to 40 |  |
| 41 to 60 |  |
| 61 to 80 |  |
| 81 to 100 |  |
| 101 to 120 |  |
| 121 to 140 |  |
| 141 to 160 |  |
| 181 to 200 |  |

# SECTION C

Please **CIRCLE** the degree to which you agree with the following statements about

# ENVIRONMENTAL ATTITUDE of ENVIRONMENTAL RESPONSIVE BEHAVIOUR.

| **Strongly Disagree**  **1** | **Disagree 2** | **Slightly Disagree**  **3** | **Slightly Agree**  **4** | **Agree 5** | **Strongly Agree**  **6** |
| --- | --- | --- | --- | --- | --- |

| 1 | I really like going on trips to the countryside, for example to forests or fields. | 1 | 2 | 3 | 4 | 5 | 6 |
| --- | --- | --- | --- | --- | --- | --- | --- |
| 2 | Governments should control the rate of raw materials usage to ensure that they last as long as possible. | 1 | 2 | 3 | 4 | 5 | 6 |
| 3 | I would like to join and actively participate in an environmentalist group. | 1 | 2 | 3 | 4 | 5 | 6 |
| 4 | I would get involved in an environmentalist organization. | 1 | 2 | 3 | 4 | 5 | 6 |
| 5 | Protecting the environment is more important than protecting peoples' jobs. | 1 | 2 | 3 | 4 | 5 | 6 |
| 6 | It makes me sad to see forests cleared for agriculture. | 1 | 2 | 3 | 4 | 5 | 6 |
| 7 | I am the kind of person who makes efforts to conserve natural resources. | 1 | 2 | 3 | 4 | 5 | 6 |
| 8 | It’s make me sad to see natural environments destroyed. | 1 | 2 | 3 | 4 | 5 | 6 |
| 9 | I do believe that the environment has been severely abused by humans. | 1 | 2 | 3 | 4 | 5 | 6 |
| 10 | Whenever possible, I try to save natural resources. | 1 | 2 | 3 | 4 | 5 | 6 |
| 11 | I think spending time in nature is entertaining. | 1 | 2 | 3 | 4 | 5 | 6 |
| 12 | I am support to governments controlling and regulating the way raw materials are used in order to try and make them last longer. | 1 | 2 | 3 | 4 | 5 | 6 |

Please **CIRCLE** the degree to which you agree with the following statements about

# ENVIRONMENTAL SUBJECTIVE NORM of ENVIRONMENT RESPONSIVE BEHAVIOUR.

| **Strongly Disagree**  **1** | **Disagree 2** | **Slightly Disagree**  **3** | **Slightly Agree**  **4** | **Agree 5** | **Strongly Agree**  **6** |
| --- | --- | --- | --- | --- | --- |

| 1 | Most people who are important to me think I should protect the environment. | 1 | 2 | 3 | 4 | 5 | 6 |
| --- | --- | --- | --- | --- | --- | --- | --- |
| 2 | Most people who are important to me want me to be environmentally friendly. | 1 | 2 | 3 | 4 | 5 | 6 |
| 3 | Most people whose opinion I value think that it is important to reduce waste. | 1 | 2 | 3 | 4 | 5 | 6 |
| 4 | Most people who I respect and admire engage in environmentally friendly behaviours. | 1 | 2 | 3 | 4 | 5 | 6 |
| 5 | Most people who are important to me protect the environment. | 1 | 2 | 3 | 4 | 5 | 6 |
| 6 | It is expected of me to be environmentally friendly. | 1 | 2 | 3 | 4 | 5 | 6 |
| 7 | I feel under social pressure to preserve the environment. | 1 | 2 | 3 | 4 | 5 | 6 |
| 8 | Most people who I admire engage in the protection of the environment. | 1 | 2 | 3 | 4 | 5 | 6 |

Please **CIRCLE** the degree to which you agree with the following statements about **ENVIRONMENTAL PERCEIVE BEHAVIOURAL CONTROL** of **ENVIRONMENT RESPONSIVE BEHAVIOUR**.

| **Strongly Disagree**  **1** | **Disagree 2** | **Slightly Disagree**  **3** | **Slightly Agree**  **4** | **Agree 5** | **Strongly Agree**  **6** |
| --- | --- | --- | --- | --- | --- |

| 1 | I find it easy to be friendly with the environment. | 1 | 2 | 3 | 4 | 5 | 6 |
| --- | --- | --- | --- | --- | --- | --- | --- |
| 2 | I find it easy to preserve resources and recycle. | 1 | 2 | 3 | 4 | 5 | 6 |
| 3 | I am confident that I can protect the environment. | 1 | 2 | 3 | 4 | 5 | 6 |
| 4 | I am fully capable of protecting the environment. | 1 | 2 | 3 | 4 | 5 | 6 |
| 5 | Thanks to my resourcefulness, I always find a way to be friendly with the environment. | 1 | 2 | 3 | 4 | 5 | 6 |
| 6 | I am in full control of my actions to protect the environment. | 1 | 2 | 3 | 4 | 5 | 6 |
| 7 | I am good at leading a green lifestyle. | 1 | 2 | 3 | 4 | 5 | 6 |
| 8 | It is easy for me to stick to my sustainability goals and preserve the environment. | 1 | 2 | 3 | 4 | 5 | 6 |
| 9 | Being friendly with the environment is in of my hands. | 1 | 2 | 3 | 4 | 5 | 6 |

Please **CIRCLE** the degree to which you agree with the following statements about of

# ENVIRONMENTAL RESPONSIVE BEHAVIOUR.

| **Strongly Disagree**  **1** | **Disagree 2** | **Slightly Disagree**  **3** | **Slightly Agree**  **4** | **Agree 5** | **Strongly Agree**  **6** |
| --- | --- | --- | --- | --- | --- |

| 1 | I Recycle. | 1 | 2 | 3 | 4 | 5 | 6 |
| --- | --- | --- | --- | --- | --- | --- | --- |
| 2 | I discuss environmental protection issues with friends and relatives. | 1 | 2 | 3 | 4 | 5 | 6 |
| 3 | I bring my own shopping bag to grocery stores. | 1 | 2 | 3 | 4 | 5 | 6 |
| 4 | I save and reuse plastic shopping bags. | 1 | 2 | 3 | 4 | 5 | 6 |
| 5 | I make a monetary donation to an environmental protection cause. | 1 | 2 | 3 | 4 | 5 | 6 |
| 6 | I actively pay attention to environmental protection and information in the media. | 1 | 2 | 3 | 4 | 5 | 6 |
| 7 | I actively participate in environmental campaigns sponsored by government and workplace. | 1 | 2 | 3 | 4 | 5 | 6 |
| 8 | I actively participate in environmental protection activities sponsored by non-governmental environmental organizations. | 1 | 2 | 3 | 4 | 5 | 6 |
| 9 | I maintain public woods and grasslands with my own money. | 1 | 2 | 3 | 4 | 5 | 6 |

***THANK YOU VERY MUCH FOR YOUR KIND COOPERATION.***
